# Pregnancy is accompanied by larger high density lipoprotein particles and compositionally distinct subspecies

**DOI:** 10.1016/j.jlr.2021.100107

**Published:** 2021-08-17

**Authors:** John T. Melchior, Debi K. Swertfeger, Jamie Morris, Scott E. Street, Carri R. Warshak, Jeffrey A. Welge, Alan T. Remaley, Janet M. Catov, W. Sean Davidson, Laura A. Woollett

**Affiliations:** 1 Department of Pathology and Laboratory Medicine, University of Cincinnati Medical School, Cincinnati, OH, USA; 2 Biological Sciences Division, Pacific Northwest National Laboratory, Richland, WA, USA; 3 Division of Biomedical Informatics, Children's Hospital Research Foundation, Cincinnati, OH, USA; 4 Department of Obstetrics and Gynecology, University of Cincinnati Medical School, Cincinnati, OH, USA; 5 Department of Psychiatry and Behavioral Neuroscience, University of Cincinnati Medical School, Cincinnati, OH, USA; 6 Lipoprotein Metabolism Section, Cardio-Pulmonary Branch, National Heart, Lung, and Blood Institute, National Institutes of Health, Bethesda, MD, USA; 7 Department of Obstetrics, Gynecology and Reproductive Sciences, University of Pittsburgh School of Medicine and Magee Women's Research Institute, Pittsburgh, PA, USA

**Keywords:** dyslipidemia, nuclear magnetic resonance spectroscopy, lipoprotein, proteomics, mass spectrometry, pregnancy, lipoprotein size, lipoprotein metabolism, phospholipids, LC-MS, AGT, angiotensinogen, ALB, albumin, APOA4, apolipoprotein A-IV, CFB, complement factor B, HDL-C, high density lipoprotein-cholesterol, LFQ, label-free quantification, PZP, pregnancy zone protein

## Abstract

Pregnancy is accompanied by significant physiological changes, which can impact the health and development of the fetus and mother. Pregnancy-induced changes in plasma lipoproteins are well documented, with modest to no impact observed on the generic measure of high density lipoprotein (HDL) cholesterol. However, the impact of pregnancy on the concentration and composition of HDL subspecies has not been examined in depth. In this prospective study, we collected plasma from 24 nonpregnant and 19 pregnant women in their second trimester. Using nuclear magnetic resonance (NMR), we quantified 11 different lipoprotein subspecies from plasma by size, including three in the HDL class. We observed an increase in the number of larger HDL particles in pregnant women, which were confirmed by tracking phospholipids across lipoproteins using high-resolution gel-filtration chromatography. Using liquid chromatography-mass spectrometry (LC-MS), we identified 87 lipid-associated proteins across size-speciated fractions. We report drastic shifts in multiple protein clusters across different HDL size fractions in pregnant females compared with nonpregnant controls that have major implications on HDL function. These findings significantly elevate our understanding of how changes in lipoprotein metabolism during pregnancy could impact the health of both the fetus and the mother.

A successful pregnancy is defined by a gestational length of 39–40 weeks with birthweights of 2.5–4.0 kg and no complications (1, 2). During pregnancy, the maternal body undergoes numerous physiological changes to ensure optimal conditions for healthy fetal growth and development (3). For example, there are dramatic shifts in plasma lipid homeostasis with major elevations in maternal plasma triglyceride and cholesterol levels (3, 4, 5) that can increase upward of 50% in this relatively short time span. Circulating lipoproteins are the vehicles responsible for distributing triglyceride and cholesterol to maternal tissues and the fetus through the placenta (6, 7, 8, 9, 10). Increased circulating levels of triglyceride and cholesterol, specifically cholesterol carried by low density lipoproteins (LDL), are typically associated with increased risks for development of coronary heart disease; however, these increases are considered healthy during pregnancy. The role of high density lipoproteins (HDL) in pregnancy is less clear and often overlooked as HDL-cholesterol (HDL-C) levels are unchanged in most pregnancies.

Lipoproteins are mostly studied for their role in lipid transport and maintenance of tissue lipid homeostasis and thus are usually defined based on measures of triglyceride and cholesterol content. Recent studies have shown that specific subclasses of lipoproteins, primarily of HDL, participate in a number of other critical physiological functions, many of which are involved in ensuring a healthy pregnancy. HDL is now appreciated for its role in regulation of lipid metabolism, hemostasis, immune response, inflammation, complement activation, metal binding, and vitamin transport (11). In addition to this functional diversity, HDL is also highly compositionally heterogeneous with over 200 different proteins potentially associated with these particles (https://homepages.uc.edu/∼davidswm/HDLproteome.html, Davidson, WS). Not surprisingly, the documented function of many of these HDL-associated proteins overlaps with many of the aforementioned functional pathways of HDL. Indeed, we have shown that these proteins are in disequilibrium on plasma HDL and tend to segregate into compositional subspecies (12, 13, 14, 15, 16), which likely perform distinct functions.

Most studies on HDL in pregnancy have focused on HDL-C, which lack the information on the changes associated with HDL subspecies and function. The goal of this study was to examine changes in lipoprotein subspecies between pregnant and nonpregnant women. We used nuclear magnetic resonance (NMR) spectroscopy and liquid chromatography-mass spectrometry (LC-MS) to characterize changes in the lipoprotein number, size, and composition. We observed profound shifts in the distribution of HDL subspecies as well as the proteomic signature in pregnant women compared with nonpregnant controls. These findings significantly aid our current understanding of lipoprotein metabolism during pregnancy and suggest that more detailed compositional studies on lipoprotein subspecies could provide keen insights into pathologies associated with adverse outcomes such as preterm birth.

## Materials and methods

### Study demographics

Women in the current study were enrolled to determine the impact of pregnancy on lipoprotein composition. Women aged 18–40 years who were in general good health were eligible. Pregnant women were recruited from the UC Health—Hoxworth Center after their initial doctor visit; samples were collected between 18 and 24 weeks of gestation based on time elapsed since last menstrual cycle. Nonpregnant women were recruited by word of mouth and fliers placed around the UC College of Medicine. Blood (30 ml) was collected after an overnight fast, after informed consent, at the Schubert Research Center (SRC) at Cincinnati Children's Hospital Medical Center (CCHMC) using K_2_EDTA as the anticoagulant to obtain plasma immediately following blood collections. The height, weight, and birthdate of the women were self-reported (nonpregnant) or obtained from medical records (pregnant). BMI was calculated from the height and weight of the women at the time of the study. A total of 24 nonpregnant women and 19 pregnant women participated in the study. The protocol was approved by the Institutional Review Board of the University of Cincinnati and follows the Declaration of Helsinki principles.

### Lipid, apolipoprotein, lipoprotein sizing, and HDL lipid composition

NMR (Vantera® Clinical Analyzer) was used to measure lipid distribution, lipoprotein size, and both particle and apolipoprotein concentrations from plasma of nonpregnant and pregnant women as previously described (17). Particles within each lipoprotein class were further resolved to report concentrations of small, medium, and large subfractions using the LP4 algorithm (18). Total and esterified cholesterol (FUJIFILM Medical Systems USA, Inc, VA) and triglyceride (MedTest Dx, MI) concentrations were measured in HDL samples isolated by ultracentrifugation (d 1.063–1.210 g/ml) from fresh plasma as previously described (19).

### Removal of APOB-containing lipoproteins and gel filtration chromatography

In a separate analysis, APOB-containing lipoproteins were precipitated from plasma by heparin and magnesium chloride (20). Briefly, plasma from nonpregnant and pregnant women was incubated with heparin and magnesium chloride for 30 min on ice, centrifuged at 1,500*g* for 30 min, and supernatant was carefully removed for analysis. HDL size subspecies were also separated by gel filtration chromatography from a subset of three nonpregnant and three pregnant women as previously described (13). Equivalent volumes of plasma (354 μl) from each individual were applied to three superdex 200 gel filtration columns (10/300 GL; GE Healthcare) in series. Plasma was eluted in standard tris buffer (150 mM NaCl, 10 mM Tris-HCl, 1 mM EDTA, 0.02% Azide) at 0.3 ml/min and 1.5 ml fractions were collected for analysis. Phospholipid elution patterns were generated by measuring lipid concentrations in each lipoprotein fraction (Phospholipids C and Cholesterol E, Wako).

### Preparation and analysis of samples by LC-MS

APOB-depleted plasma and the HDL subspecies in each fraction spanning fractions 20–30 were incubated with calcium silicate hydrate (lipid-removal agent, LRA) to sequester lipid-associated proteins as previously described (13, 20). The bound lipoproteins were washed thoroughly with 10 mM ammonium bicarbonate (pH = 8.0), and proteins were digested directly off the LRA by incubation with sequencing-grade trypsin overnight at 37 °C with gentle shaking. Digested peptides were collected and reduced with 10 mM DTT for 30 min at 42 °C followed by alkylation with 40 mM iodoacetamide for 30 min at room temperature in the dark. Peptides were dried under vacuum and stored at –20 °C until ready for analysis by LC-MS. Peptides were reconstituted in 15 μl solvent A (formic acid/H_2_O 0.1/99.9, v/v) and 5 μl was loaded onto a reverse phase column (ACQUITY UPLC C18, Waters) maintained at 40 °C with an Infinity 1290 autosampler and HPLC (Agilent). Peptides were eluted at 0.1 ml/min using a mobile phase gradient from 5% solvent B (formic acid/acetonitrile 0.1/99.9, v/v) to 32% solvent B over 60 min to 50% solvent B over 2 min. The column was cleaned by ramping the mobile phase gradient to 90% solvent B for 10 min followed by re-equilibration of the column at 5% solvent B for 10 min. Peptides were introduced to the mass spectrometer using a Jet Stream source (Agilent) and identified and fragmented as previously described (21). Precursors were limited to acquisition of ions with charge states of 2+ and 3+ and required a minimum of 1,500 counts. Each cycle acquired the 20 most intense precursors, which were fragmented with a variable collision energy (CE) dependent on the precursor mass-to-charge (m/z) ratio: CE = k∗ (m/z) + b, with a slope (k) of 3 and an offset (b) of 2 for 2+ ions and −2 for 3+ ions. MS/MS spectra were acquired until at least 45,000 total counts were collected or a maximum accumulation time of 0.33 s.

### Mass spectrometry data analysis

The LC-MS data for all samples were analyzed using the bioinformatic pipeline shown in supplemental Fig. S1. First, mascot generic files (MGFs) were generated from the raw data files using MassHunter Qualitative Analysis Software (v B.07.00, Agilent). MS/MS peaks were limited to the top 150 peaks by height and precursors were limited to a maximum assigned charge state of 3+. Peptide spectral data was matched against the UniProtKP/Swiss-prot Protein knowledgebase for Homo sapiens (version: release 2017_11, date: 12-20-2017, entries: 20,319) using the Mascot search engine (v 2.2.07). Trypsin was used to generate peptides and data was constrained to allow for a maximum of three missed cleavages, fixed modification of carbamidomethylation, and a variable modification of oxidation with a peptide tolerance of 35 ppm and MS/MS tolerance of 0.6 Da. Scaffold (v4.3.4) was employed for MS/MS peptide validation using X! Tandem (2010.12.01.1, subset of 1,590 entries). Identified proteins and peptides were constrained to 99.9% and 95.0% probability, respectively, and protein inclusion required identification of at least two unique peptides. The list of proteins was further constrained to only including proteins that showed up in the same fraction across all three patients within an experimental cohort. For example, protein A had to meet the minimum identification thresholds in fraction N of either all three nonpregnant or all three pregnant females to be included in the list. A final list of 92 proteins were selected and integrated into a FASTA file. As raw Agilent files are incompatible with MaxQuant, the Agilent data files were first formatted to mzML files with 32-bit binary encoding precision, write index, and TPP compatibility for all MS levels using the Vendor Algorithm. The mzML files were further formatted into mzXML files with forced MaxQuant compatibility using TOPPAS file converter. The final mzXML files and custom fasta file were used to perform label-free quantification (LFQ) using MaxQuant (22). Samples were further processed by exporting the final proteomic LFQ data into Perseus (23) for final analysis.

### Cholesterol efflux

Cholesterol efflux was measured using APOB-depleted plasma as described (24) with minor modifications. Briefly, RAW 264.7 macrophages were grown to confluency and incubated with media containing 1.0 μCi/ml [1,2–^3^H(N)]-cholesterol. After extensive washing, media containing equal volumes of sample (40 μl) ±0.3 mM 8-bromo-cAMP were incubated with the macrophages for 8 h. In all experiments, media alone and media containing 10 μg/ml of plasma APOA1 by protein were included as quality controls. Cholesterol efflux was measured by harvesting the media, filtering with a 0.45 μm filter, and quantifying radiolabeled cholesterol by scintillation counting. Each subfraction from each donor was run in triplicate, and the percent efflux was calculated by dividing the number of counts in the media by the total internalized counts per well (determined from the media-only treated cells). Data are presented on an equal volume basis and where stated, the percent efflux was normalized by total phospholipid concentration of the APOB-depleted samples measured enzymatically.

### Statistics

Data are presented as averages ± SEM. Demographics (maternal age, maternal BMI) were analyzed by student's t-tests. Sizing for each lipoprotein was analyzed using two-way repeated measures ANOVA (between-subjects factor is pregnancy and within-subject factor is particle size) using BMI as an interacting covariate (*i.e.*, effects of pregnancy on the particle size distribution were allowed to possibly depend on BMI). We analyzed lipid and apolipoprotein concentrations by one-way ANOVA with BMI as a covariate. Proteomic differences were determined in total HDL by ANOVA with multiple-testing adjustment to achieve a maximum false discovery rate (FDR) of 5% to generate a *q*-value. FDR-adjusted significance levels were computed for pairwise comparisons for all proteins, and we note those that met the FDR-corrected threshold (*q* < 0.05).

## Results

### Demographics and plasma lipids

Nonpregnant and pregnant women were similar in age with pregnant women having greater BMI than nonpregnant women (Table 1, supplemental Fig. S2). Body weights of pregnant women were obtained at the time of blood draw, which ranged from 18 to 24 weeks gestation. A summary of the measures from the standard lipid panel in nonpregnant and pregnant women is shown in Table 1. Total cholesterol concentrations were ∼20% greater in the pregnant compared with nonpregnant controls with most of the elevation observed in concentrations of LDL-C. We observed a parallel increase in plasma APOB concentrations of pregnant women with no significant differences in plasma triglyceride or triglyceride-rich lipoprotein (TRL)-C concentrations. Taken together, this suggests that the elevation in total cholesterol during pregnancy was due to increased number of LDL particles as opposed to increased cholesterol content of the LDL fraction. Indeed, there were strong correlations between APOB and LDL-C concentrations (*r* = 0.95, *P* < 0.0001) and APOB and plasma cholesterol concentrations (*r* = 0.93, *P* < 0.001). Conversely, plasma HDL and APOA1 concentrations were similar between groups. There was no relationship between any of these parameters and BMI, with BMI as either a main effect or as an interaction with pregnancy.

**Table 1:** Clinical characteristics of study participants

Characteristics	Non-pregnant (N = 24)	Pregnant (N = 19)	*P*
Age (years)	31.7 ± 1.3	28.9 ± 1.3	0.153
BMI	24.7 ± 1.3	30.9 ± 1.7∗	**0.004**
Gestational age (weeks)		21.7 ± 0.7	
Plasma cholesterol (mg/dl)	188.1 ± 7.2	227.4 ± 12.4∗	**0.039**
TRL-C (mg/dl)	18.9 ± 1.9	23.6 ± 2.8	0.366
LDL-C (mg/dl)	103.7 ± 5.4	134.3 ± 10.5∗	**0.017**
HDL-C (mg/dl)	65.5 ± 3.1	69.6 ± 2.9	0.892
Plasma triglyceride (mg/dl)	83.8 ± 9.0	94.0 ± 7.3	0.207
APOB (mg/dl)	77.7 ± 4.3	100.2 ± 6.5∗	**0.022**
APOA1 (mg/dl)	157.2 ± 4.8	168.8 ± 6.0	0.750

Abbreviations: APOA1, apolipoprotein A-I; APOB, apolipoprotein-B100; BMI, body mass index; HDL-C, high density lipoprotein cholesterol; LDL-C, low density lipoprotein cholesterol; TRL-C, triglyceride-rich lipoprotein cholesterol.

Values represent mean ± standard error of mean.

*P*-values derived by Student's *t*-tests (demographics) and lipids and apolipoproteins by one-way ANOVA with BMI as a covariate. Bold represents significant differences.

### Lipoprotein size and particle number

Recent studies have shown that advanced lipid analysis of lipoprotein subspecies using NMR spectroscopy can provide additional insights into individual health beyond those of standard plasma lipid panels (25, 26, 27). Using NMR spectroscopy and the LP4 algorithm, we performed an analysis of lipoprotein subspecies in the plasma of nonpregnant and pregnant women (Table 2). The sizes of TRL and LDL were similar in all women while plasma HDL in pregnant women was significantly larger compared with nonpregnant women.

**Table 2:** Size and concentration of lipoprotein subspecies

Subfraction	Diameter Range (nm)	Diameter		Concentration	
		NP	P	NP	P
TRLP	24–240	45.6 ± 1.4	46.0 ± 1.6	103.4 ± 10.1	11140.6 ± 17.8
Very small	24–29			52.2 ± 7.7	91.3 ± 14.4∗∗∗
Small	30–36			36.4 ± 7.2	30.3 ± 6.8
Medium	37–49			12.6 ± 1.9	16.8 ± 2.9
Large	50–89			2.1 ± 0.7	2.0 ± 0.6
Very large	90–240			0.1 ± 0.0	0.1 ± 0.0.0
LDLP	19–23	21.1 ± 0.1	20.9 ± 0.1	1356.0 ± 65.3	1807.4 ± 116.1∗∗∗
Small	19–20			647.3 ± 74.1	1292.1 ± 84.0∗∗
Medium	20–21			300.2 ± 73.3	120.7 ± 71.5
Large	21–23			408.5 ± 46.5	394.7 ± 80.5
HDLP	7.5–13	9.4 ± 0.1	9.7 ± 0.1∗	22.8 ± 0.5	22.0 ± 0.5
Small	7.4–7.8			12.9 ± 0.6	13.8 ± 0.6
Medium	8.7–9.5			6.3 ± 0.6	2.1 ± 0.4∗∗∗
Large	10–12			3.6 ± 0.5	6.1 ± 0.4∗∗

Abbreviations: HDP, high density lipoprotein particle; LDLP, low density lipoprotein particle; NP, nonpregnant; P, pregnant; TRLP, triglyceride-rich lipoprotein particle.

Concentration is reported at nmol/L for TRLP and LDLP and μmol/L for HDLP.

Values represent mean ± standard error of mean of 24 and 19 individuals in the nonpregnant and pregnant groups, respectively.

Asterisks denote statistically significant differences at ∗*P* < 0.05; ∗∗*P* < 0.01; ∗∗∗*P* < 0.001.

**Fig. 1:**
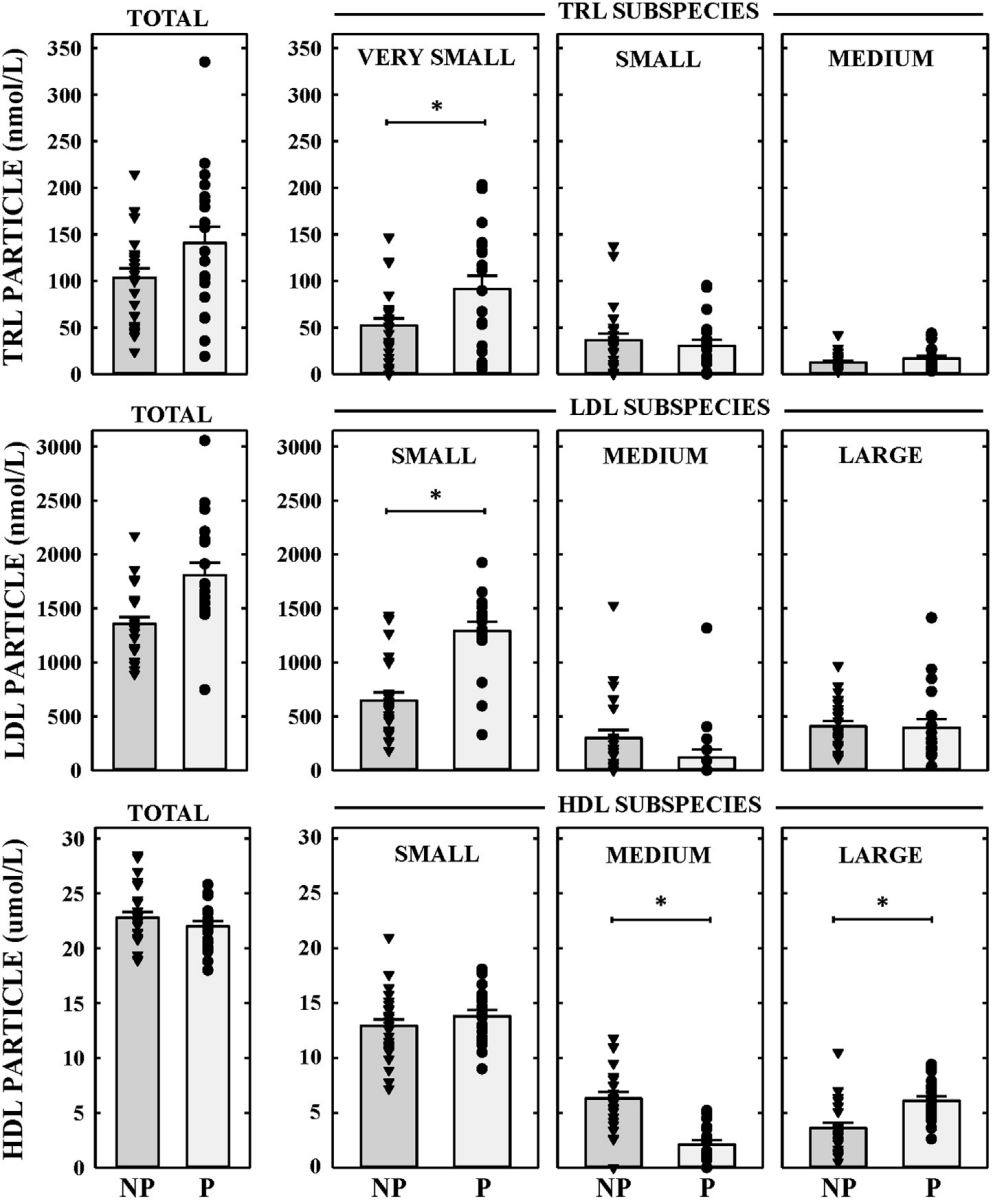
**Impact of pregnancy on lipoprotein subspecies**. Lipoprotein particle concentrations (μmol/L) in nonpregnant (NP) and pregnant (P) women. Total TRL particles and TRL subspecies (very small, small, and medium particles) concentrations are shown in the *upper panel*. Total LDL particles and LDL subspecies (small, medium, and large particles) concentrations are shown in the *middle panel*. Total HDL particles and HDL subspecies (small, medium, and large particles) concentrations (μmol/L) are shown in the *bottom panel*. Each data point represents a single female; some data points represent more than one woman if values are the same, especially when zero. ∗ represents significant differences (*P* < 0.05) between nonpregnant and pregnant women.

The lipoprotein populations were further subdivided into size-defined particle subclasses as previously reported (28). One of the subspecies measured are classified as TRL, which encompass a broad range of traditional larger-sized lipoprotein subclasses such intermediate density lipoproteins (IDL, 25–35 nm), VLDL (30–80 nm), and even some chylomicrons (75–1,200 nm), though these women were fasted. We found total TRL particle concentrations were similar between both groups of females (Fig. 1, top). The very small particles accounted for a majority of the TRL fraction, and there was a 40% increase in the very small particles in the pregnant compared with nonpregnant females. There were no differences in the amounts of small and medium particles between groups. Concentrations of large and very large TRL particles were very low and not different between the nonpregnant and pregnant females.

The concentration of LDL particles was the highest among the APOB-containing lipoproteins measured (Fig. 1), which is reflected by its strong correlation to APOB concentration (*r* = 0.97, *P* < 0.001). Plasma LDL particles were significantly higher in pregnant females (Fig. 1, middle), which were driven primarily by the increase the amount of small LDL particles, which were 2-fold higher in pregnant women compared with their nonpregnant counterparts. There was no difference in concentrations of medium or large LDL particles between nonpregnant and pregnant females though medium particles trended toward being decreased in pregnant females (*P* = 0.088).

Plasma HDL is the most heterogeneous of the lipoprotein subfractions. The concentration of total HDL particles was the highest among all the lipoprotein subclasses with the number of HDL particles over 10-fold higher than LDL and TRL combined (note HDLP is reported as μmol/L). Similar to plasma HDL-C, no differences were observed in HDL particle number between nonpregnant and pregnant females (Fig. 1, bottom). Most of the HDL in both groups was comprised of small particles, which were no different between groups. In contrast, pregnant females exhibited significantly less medium HDLs and more large HDLs compared with nonpregnant women.

### HDL speciation and composition

**Fig. 2:**
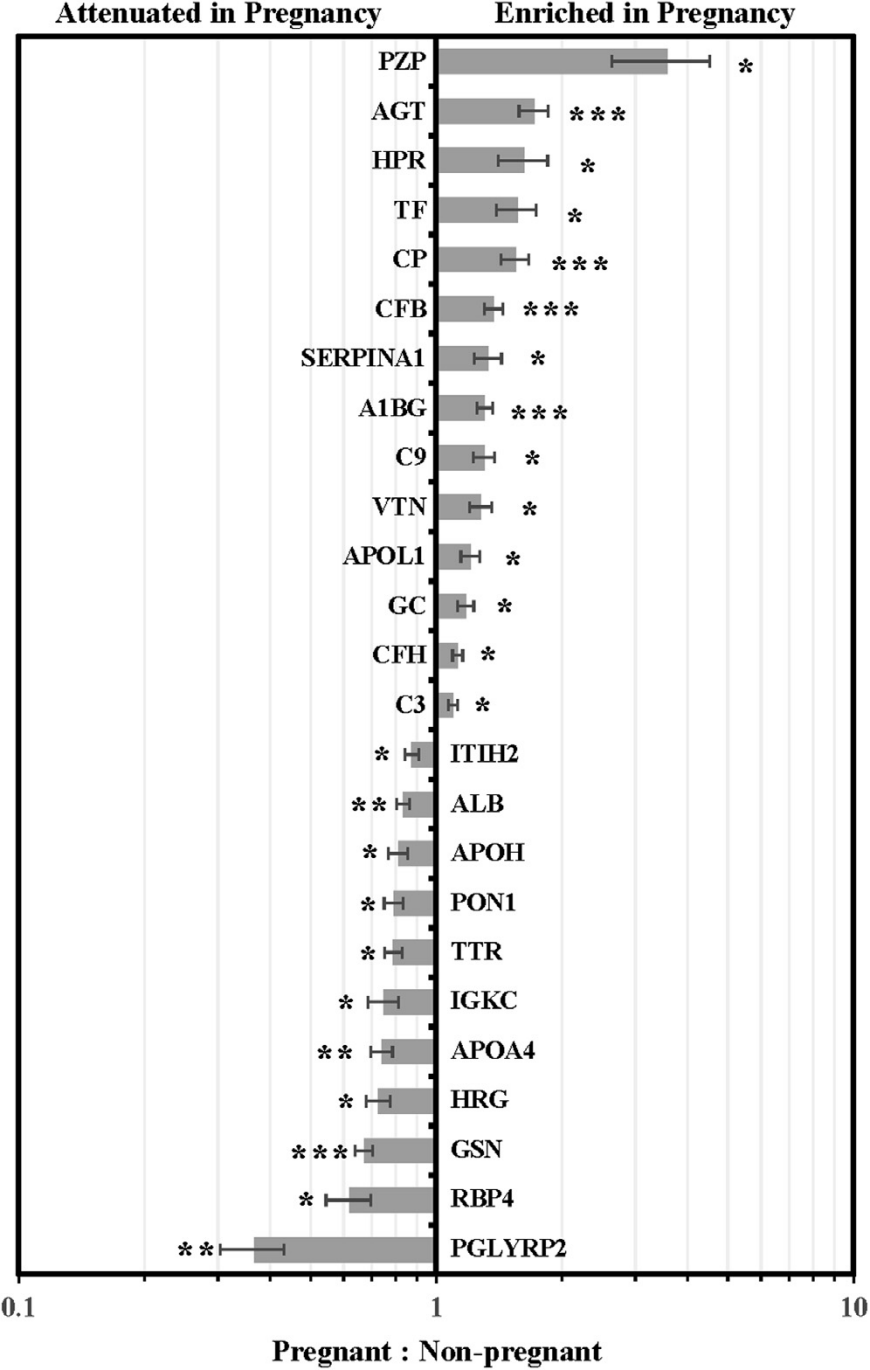
**Impact of pregnancy on the HDL proteome**. HDL was isolated by APOB-depletion and binding to LRA. Proteins were analyzed by LC-MS and abundance quantified using MaxQuant as described. Bars represent the ratio of the mean abundance of the protein in pregnant (N = 19) to nonpregnant women (N = 24) and error bars represent standard error across samples. *Asterisks* denote level of significance at ∗*P* < 0.05, ∗∗*P* < 0.01 or ∗∗∗*P* < 0.001.

The compositional diversity of HDL with regard to their proteomic signature has garnered significant attention as of late (16, 20, 29, 30). We investigated compositional differences in the APOB-depleted plasma from nonpregnant and pregnant females by identifying and quantifying relative protein abundance using LC-MS and MaxQuant (22). Plasma was first depleted of APOB-containing lipoproteins, then the remaining lipid-associated complexes were adsorbed to a phospholipid binding resin to eliminate free proteins. Despite this material being composed almost entirely of HDL, we are careful to refer to this as “APOB-depleted plasma” and not “HDL” as it technically contains a small amount of other lipid-containing vesicles. Using shotgun proteomics we identified 70 total proteins across nonpregnant and pregnant females; no unique proteins were found in either group. All of the identified proteins in the current study have been previously reported to be associated with HDL based on the HDL proteome watch (https://homepages.uc.edu/∼davidswm/HDLproteome.html, Davidson, WS). We went on to perform LFQ of the identified proteins to determine if pregnancy had any impact on their relative abundance in the APOB-depleted samples. We identified significant changes in 25 HDL-associated proteins, which are expressed as fold difference in LFQ intensity in pregnant *versus* nonpregnant females in Fig. 2. A total of 14 proteins were enriched in pregnant females with the biggest and most significant differences in pregnancy zone protein (PZP), angiotensinogen (AGT), ceruloplasmin (CP), complement factor B (CFB), and alpha-1B-glycoprotein (A1BG). Conversely, we observed a decrease in 11 proteins in pregnant females with the largest differences or most significant differences in N-acetylmuramoyl-L-alanine amidase (PGLYRP2), apolipoprotein A-IV (APOA4), and albumin (ALB). A full list of proteins and LFQ intensities for the APOB-depleted samples can be found in supplemental Table S1.

**Fig. 3:**
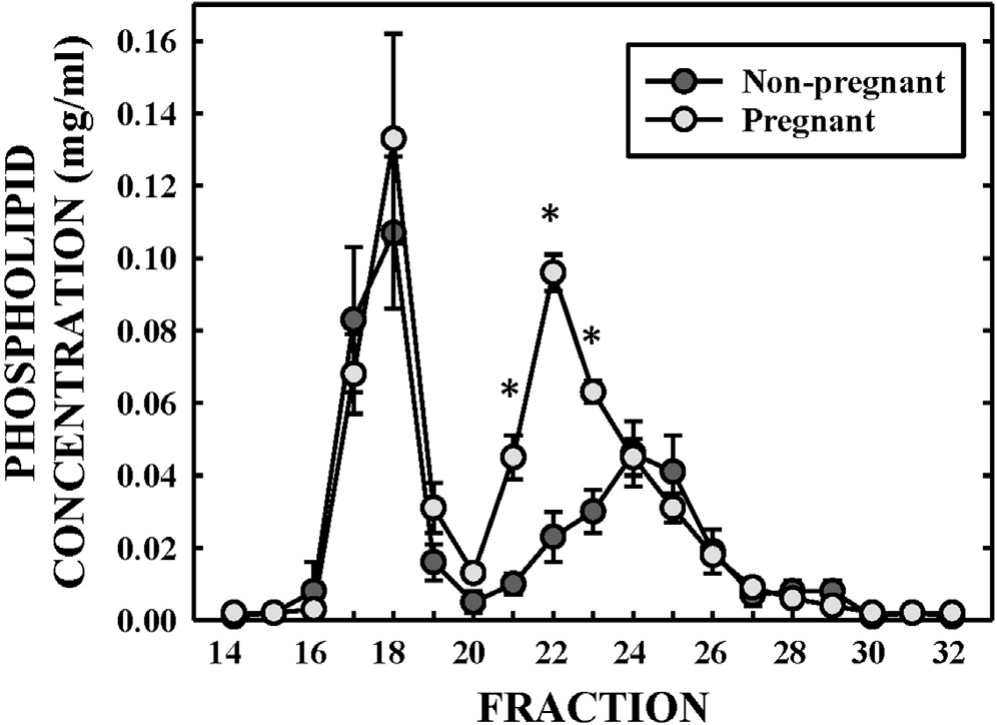
**Impact of pregnancy on lipoprotein distribution**. Plasma from nonpregnant and pregnant females was separated using gel filtration chromatography. Phospholipid concentration was measured in each fraction. Based on previously published information (86), the TRL/LDL particles (fractions 15–19) were 19–240 nm, large HDL particles (fractions 20–22) were 10.2–12 nm, medium HDL particles (fractions 23–24) were 8.7–10.2 nm, and small HDL particles (fractions 25–26) were 7.4–8.6 nm. Fractions 27–30 contained lipid-poor proteins. Bars represent standard deviation of n = 3 patients in each group. ∗ denote statistically significant differences between nonpregnant and pregnant groups at *P* < 0.05 as determined by Student's *t* test.

We next investigated whether the proteomic differences observed in the total HDL between nonpregnant and pregnant females were specific to distinct HDL subspecies. As the NMR technique for quantitating lipoprotein subspecies is solely analytical, we utilized an orthogonal approach for separating lipoproteins by size using high-resolution gel filtration chromatography. This separation has the added advantage of providing material for additional biochemical characterizations of particle composition. Whole plasma was applied to three superdex 200 columns in series, which results in a broad HDL peak that resolves different HDL subspecies (fractions 20–30) and a single peak where VLDL and LDL coelute between fractions 15 and 19. The isolations were performed on a subset of three nonpregnant and three pregnant females with matched BMI. The HDL-sized fractions were collected and subjected to LC-MS analysis after isolating lipid-containing species from comigrating plasma proteins by LRA (see Methods) (13). Figure 3 shows the phospholipid distribution across the plasma lipoproteins. We observed a more dramatic increase in the large-sized HDL particles (fractions 20–22) in pregnant women compared with NMR measures. Nonpregnant women showed primarily medium-sized particles (fractions 23–25) though the increase compared with pregnant women was more modest using gel filtration chromatography. These seemingly disparate measures between techniques likely result from the different entities being measured; NMR quantifies particle number based on the methyl groups on lipids to give a total while phospholipid concentration is determined on the fractionated plasma isolated using gel filtration. In the latter case, phospholipid values can represent an enrichment of phospholipid in the particles and/or an increase in the number of phospholipid-containing particles in the fraction.

**Fig. 4:**
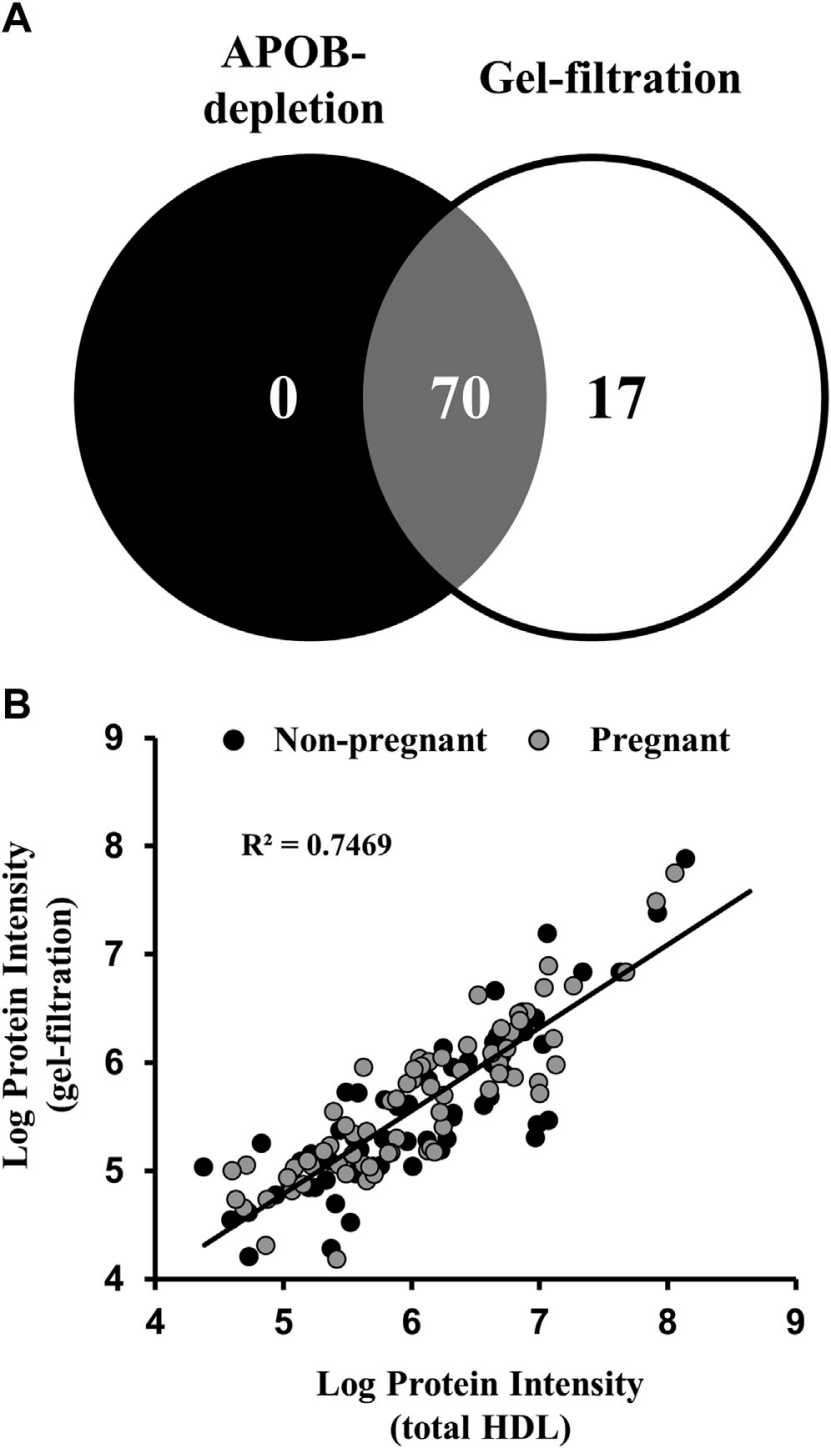
**Comparison of HDL-associated proteins identified in total and speciated HDL**. Proteins were identified by LC-MS as described. *A*, Venn diagram showing the distribution of identified proteins in HDL isolated by APOB depletion (*black*) and HDL speciated by gel filtration chromatography (*white*). *B*, scatterplot showing relationship between shared proteins identified in total HDL and speciated HDL. Data points represent log protein abundance for each protein. Protein abundance in total HDL was determined as the mean of the LFQ intensities across each group (N = 19 and 24 in nonpregnant and pregnant females, respectively). Protein abundance in the speciated HDL was determined by summing the protein LFQ intensities across all fractions and determining the mean across the three individuals in each group.

We went on to perform proteomic analysis on lipid-containing particles spanning the HDL-size range (fractions 20–30). In total (*i.e.*, all fractions considered together), we found that speciation of HDL by gel filtration resulted in the identification of 17 additional unique proteins that were not identified in total plasma samples subjected to APOB-depletion described above (Fig. 4A). Nonetheless, when we examined the relationship between the common proteins identified between the two methods, we observed an excellent correlation in protein abundance between the two isolation procedures (Fig. 4B). This supports the notion that the subset of females randomly selected from each group is highly representative of the larger clinical cohort. The LFQ intensities of each protein identified across gel filtration fractions were imported into Perseus, which was used to determine the protein z-score. The z-score is a normalization reflective of the number of standard deviations above or below the mean of a given protein across all fractions in both the nonpregnant and pregnant females. A hierarchical clustering of proteins was performed based on the Euclidian distance between the averages of the protein abundance pattern across the fractions.

**Fig. 5:**
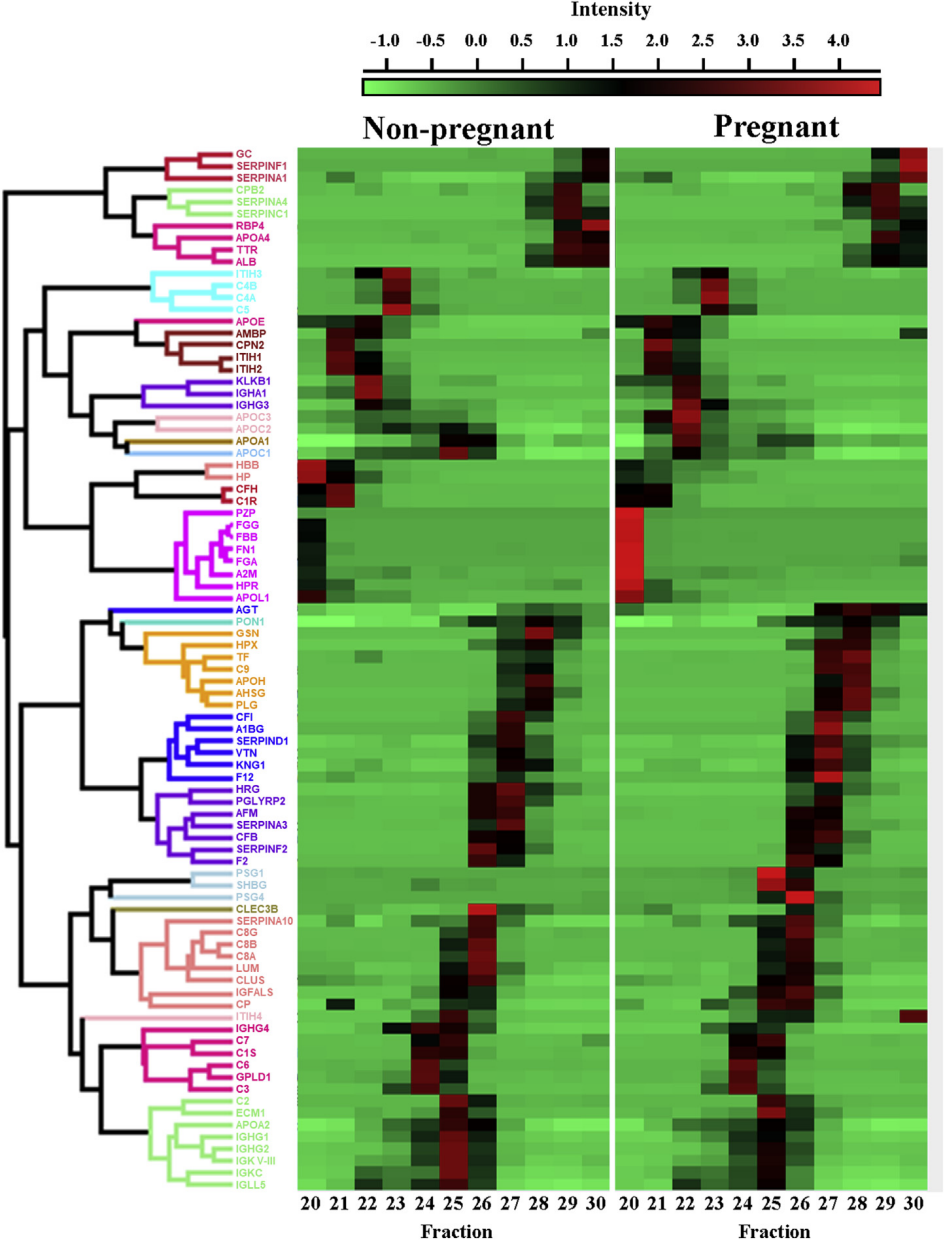
**Impact of pregnancy on proteome of HDL subspecies**. Fractions 20–30 spanning the HDL-sized fractions were collected using gel filtration chromatography and analyzed by LC-MS. Label-free quantification was performed on 87 identified proteins across proteomic experiments. Hierarchical clustering of proteins based on z-score across fractions in nonpregnant (*left*) and pregnant (*right*) individuals. Protein clusters with a Euclidean distance < 3.0 are colored in the dendogram

The protein clusters and accompanying heat map of the z-score are shown in Fig. 5 with HDL fractions in nonpregnant females on the left and pregnant females on the right. We set a relative Euclidean distance of <3.0, which resulted in 25 total protein clusters that are colored in the dendogram. We observed striking changes in the abundance of several of these clusters in specific HDL fractions in pregnant females. The most apparent increase was found in the eight-member cluster containing PZP, FGG, FBB, FN1, FGA, A2M, HPR, and APOL1 in fraction 20. We also observed more modest increases in three-member protein clusters PSG1, PSG4, and SHBG in fraction 25 and GC, SERPINF1 and SERPINA1 in fraction 30. Conversely, there were clear decreases in the two-member cluster of HBB and HP and three-member cluster of KLKB1, IGHA1, and IGHG3 in fractions 20 and 22, respectively. Decreases in larger clusters such as the eight-member cluster of C2, ECM1, APOA2, IGHG1, IGHG2, IGKV-III, IGKC, and IGLL5 in fraction 25 and four-member cluster of RBP4, APOA4, TTR, and ALB in fraction 30 were also observed. A full list of proteins and LFQ intensities for the fractionated samples can be found in supplemental Table S2.

### Cholesterol efflux

**Fig. 6:**
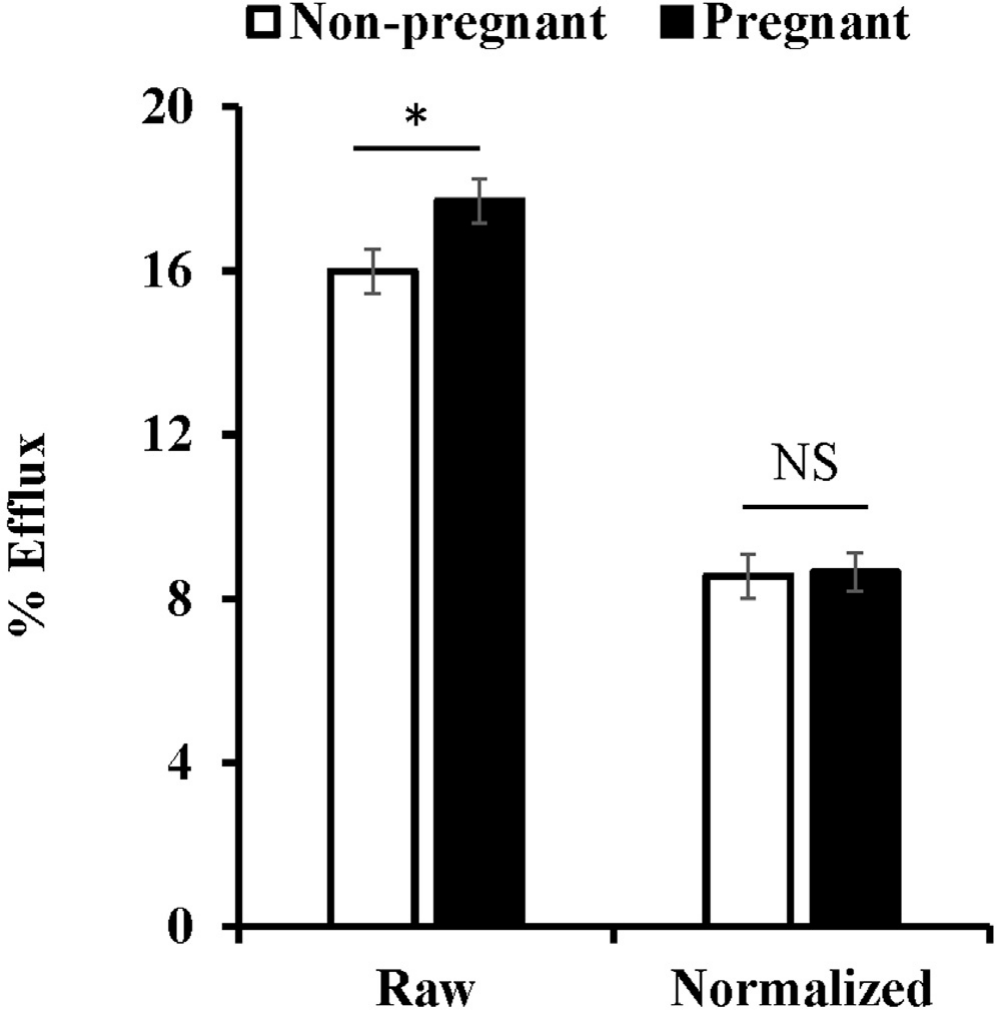
**Cholesterol efflux of** APO**B-depleted plasma from pregnant and nonpregnant women**. Plasma from pregnant and nonpregnant women was APOB-depleted and their ability to mediate cholesterol efflux was determined on RAW 364.7 macrophages treated with 8-bromo-cAMP. Cholesterol efflux was determined using equal volumes of APOB-depleted plasma (raw, *left*) and normalized to total phospholipid content in APOB-depleted fractions (normalized, *right*). Bars represent averages ± standard error for n = 22 nonpregnant women and n = 19 pregnant women. Asterisks denote level of significance at *P* < 0.05 as determined using a Tukey-Kramer HSD.

Given the striking differences in the HDL species and particle composition observed in both the APOB-depleted plasma and fractionated plasma, we wanted to determine if these differences impacted HDL function. To do this, we measured the cholesterol efflux capacity on equal volumes of APOB-depleted plasma from pregnant and nonpregnant females incubated with RAW 264.7 macrophages that were stimulated with cAMP (Fig. 6). We found that APOB-depleted plasma from pregnant subjects exhibited a modest, but significant increase capacity to efflux cholesterol from cells compared with nonpregnant counterparts. Given the differences in particle number and phospholipid concentration observed in the NMR and gel filtration analyses, we sought to determine if efflux was due to active transport (*i.e.*, ABCA1-mediated) or passive diffusion due to phospholipid differences. To do this, efflux was normalized to total phospholipid content loaded in the assay. As shown in Fig. 6, the differences in efflux between APOB-depleted plasma from nonpregnant and pregnant women were no different after normalization indicating that the observed differences in nonnormalized data are likely due to the changes in the aqueous diffusion pathway.

## Discussion

NMR, high-resolution gel filtration chromatography, and mass spectroscopy were used to examine the impact of pregnancy on lipoprotein subspecies concentrations and compositions, with a specific focus on the proteomic makeup of the particles. The impact of pregnancy on cholesterol concentrations was consistent with previous reports, which show increases in total and LDL-C but modest to no change in HDL-C. The observed 30% increase in APOB concentrations in the plasma of pregnant women is in line with past studies (31, 32, 33) and is likely due to the estradiol-induced increase of APOB production (34, 35). We found the increase was specific to very small TRL and small LDL particles in the pregnant females. The increase in these populations is thought to be due to decreased hepatic uptake due to decreased enrichment in APOE (36) and decreased affinity to the LDL receptor (37). Small, dense LDL is associated with heart disease, however, mostly because it has the greatest extent of lipid modifications, which are often proinflammatory (38). How these features may be related to pregnancy is not known. While the proteomic composition and function of these particles were out of the scope of the current study, their relationship with pregnancy warrants further investigation given their critical role in lipid transport and metabolism.

Deeper investigation of the HDL subspecies revealed that pregnancy had a profound impact on the quantity of the distinct particle subtypes; an important observation as HDLs' diverse functionality depends on both the size and proteomic composition of the particles. NMR measures show that pregnancy resulted in an increase in the number of large HDL at the expense of medium-sized particles, a shift that was more pronounced after analysis by high-resolution gel filtration chromatography. While several factors likely contribute to this increase in particle size, multiple studies have demonstrated increased triglyceride content in HDL isolated from pregnant women suggesting that changes in the neutral lipid core content could be driving the observed differences (39, 40, 41). Although the current study was focused on the proteomic composition of the particles, we did observe a trend (*P* = 0.07) toward increased triglyceride content of a subset of HDL isolated by ultracentrifugation in pregnant *versus* nonpregnant women consistent with these reports (*data not shown*). Previous studies have reported that these triglyceride-rich HDLs are associated with increased levels and activity of CETP (39, 40, 41) while others report the triglyceride enrichment results from decreased hepatic lipase activity (33, 42, 43). Interestingly, we observed an increase in APOA2 associated with HDL in larger particles in fraction 22 (Fig. 5, supplemental Table S2), which can sequester liberated hepatic lipase thereby inhibiting its activity (44, 45). We suspect these particles result from a combination of changes in several pathways as part of a programmed metabolic response designed to satisfy the energy-requiring needs of the fetus (46, 47). Indeed, multiple cell types in the placenta express receptors important for the uptake of HDL and maternal-fetal transport of some of the HDL cargo (6, 7, 47, 48, 49, 50, 51, 52).

While lipid uptake by the placenta is critical for healthy embryo development during pregnancy, too much lipid, especially sterol, in any cell or tissue can be deleterious. Here we observed that maternal HDLs exhibit a modest, but significant increase in their ability to efflux cholesterol. However, despite the large proteomic changes observed in the study, it appears this increase is due entirely to increases in the large, phospholipid-enriched particles that are present in the pregnant females. Our group has previously reported the acute appearance of large HDLs after weight loss surgery similar to those observed in pregnant women here (53). Consistent with our measures, that study showed an increase in cholesterol efflux capacity in APOB-depleted plasma from the same patients associated with the appearance of those particles. While we did not specifically measure the cholesterol efflux capacity of the large particles in the current study, previous work from our group (54) strongly indicates that the increased phospholipid content we see with the rise in the large particle population in pregnancy is likely driving the increase efflux capacity observed in the APOB-depleted plasma of pregnant women. As mentioned above, this would suggest that the rise of these particles is playing an important role in lipid homeostasis at the maternal-fetal interface.

Pregnancy also resulted in profound changes in several proteins associated with different plasma HDLs. In APOB-depleted plasma, many of the proteins enriched on HDL are known to inhibit proteases (PZP, AGT, SERPINA1) and play major roles in the complement pathway (CFB, C9, CFH, and C3). Surprisingly, proteins that were attenuated in APOB-depleted plasma of pregnant women are known to inhibit inflammation (ALB, PON1, APOA4). We also found that two trypanosome lytic factor proteins, which are constitutively innate immune effectors, APOL1 and haptoglobin-related protein (HPR) (55), were enriched in pregnant HDL. Taken together, this suggests a compositionally altered HDL in pregnancy that could potentially modulate inflammation and mediate innate immunity. This is important as pregnancy results in a complex inflammatory response where inflammation is greater in early pregnancy to allow for implantation of the fetus, but decreases mid-pregnancy to dampen the immune-modulated rejection of the semiallogeneic fetus (56). Left unchecked, inflammation can result in adverse pregnancy outcomes, including preterm birth and preeclampsia (57, 58, 59, 60, 61, 62, 63). Given the major role of HDL in the inflammatory response (64), the observed changes in these proteins suggest they may play major roles in toggling that response in pregnant women. Pregnancy also resulted in increases in proteins important in autophagy (fibrinogens, macroglobulin, and haptoglobin) and vitamin A binding and transport (TTR, RBP4), which appear mostly localized to the large HDL. Autophagy and vitamin transport have both been associated with various aspects of maintaining a healthy pregnancy at the level of the placenta and implantation and at systemic inflammation (57, 60, 65, 66, 67, 68, 69, 70).

In summary, pregnancy alters HDL particle size and a number of protein clusters on distinct HDLs. It is likely these compositional changes alter the particle function in several metabolic pathways important for maternal and fetal heath. Understanding the molecular details of HDL composition and function in normal pregnancy is critical for identifying potential alterations in HDLs associated with adverse outcomes, especially those associated with excessive inflammation, such as preterm deliveries or preeclampsia. These adverse outcomes affect about ≈10% and ≈4% of pregnancies, respectively, and have severe long- and short-term consequences for the mother and infant (71, 72). Our findings suggest that monitoring these more informative molecular markers could provide essential metabolic readouts on fetal and maternal health during pregnancy. These measures could also provide insights into the mechanisms responsible for increased development of cardiovascular disease in women with preterm deliveries or preeclampsia (73, 74, 75, 76, 77).

### Strengths and limitations

The main strength of this study was that we examined the impact of pregnancy on lipoprotein subspeciation, including concentrations and compositions of the various subspecies of HDL. Previous studies have shown rather unremarkable changes in generic markers of HDL (*i.e.*, HDL-C) likely deincentivizing deeper dives into the role of HDL in pregnancy. Our work unequivocally shows that, while overall cholesterol levels and even particle numbers do not change in HDL, the quality, and likely functionality, of those particles may shift dramatically. We suggest that this opens the door to understanding how changes in HDL functionality might impact adverse outcomes, as suggested by previous studies (33, 36, 78, 79, 80, 81). The main weakness of our study design was its cross-sectional nature. Ideally, and hopefully pursued in future studies, a longitudinal study can be done in which HDL profiling is done in women prior to, during (several time points), and after pregnancy. This would factor out inevitable differences between women allowing for temporal differences to be monitored within each subject. In addition, racial information was unavailable for most of the women in this study. It is unlikely that race will affect the results as a previous study showed no effect of race on LDL or HDL sizing, though possibly VLDL sizing, in pregnant women (81) or cohorts of nonpregnant women and men (82, 83). We also did not obtain the prepregnancy BMI and thus did not determine gestational weight gain, though excessive weight gain has not been shown to be associated with lipids in early and mid-gestation (84).

## Data availability

The mass spectrometry proteomics data have been deposited to the ProteomeXchange Consortium *via* the PRIDE (85) partner repository with the dataset identifier PXD026543 for the APOB-depleted plasma and PXD026683 for the SEC fractions.

## Supplemental data

This article contains supplemental data.

## Conflict of interest

The authors declare that they have no conflicts of interest with the contents of this article.
