# Supplementary material for: Pregnancy is accompanied by larger high density lipoprotein particles and compositionally distinct subspecies
Source: J Lipid Res. 2021 Aug 17;62:100107. doi: 10.1016/j.jlr.2021.100107 (PMC8441201; doi:10.1016/j.jlr.2021.100107)
Supplement: Supplemental Figs. S1 and S2 [file mmc1.docx]

**Supplemental Figure Legends**

**Supplemental Figure 1: Workflow for proteomic analysis of HDL and HDL subspecies as determined by LC-MS.** Raw data files were analyzed by Mascot followed by Scaffold to obtain a list of proteins and associated spectral counts. The protein list was weened by eliminating any proteins that failed to show up in the same fraction across the three patients within respective groups (pregnant or non-pregnant. That list was used to generate a FASTA file which was combined with mzXML files generated from the raw data and entered into MaxQuant to obtain label-free quantification of the proteins by MS1 abundance. Data from the MaxQuant analysis was imported into Perseus for statistical comparisons.

**Supplemental Figure 2: BMI and age of pregnant and non-pregnant women.** Averages ± standard error mean are shown for both groups of women. Each data point represents an individual.

**Supplemental Figure 1**


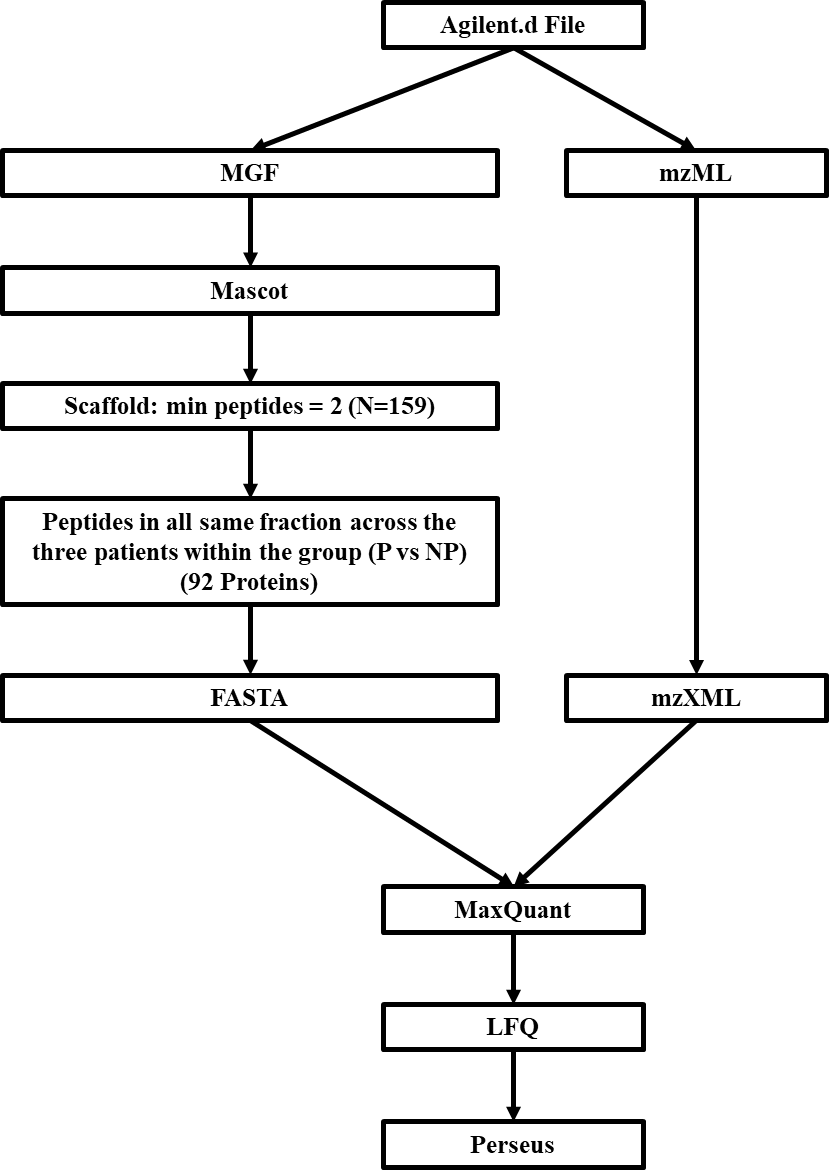


**Supplemental Figure 2**

**
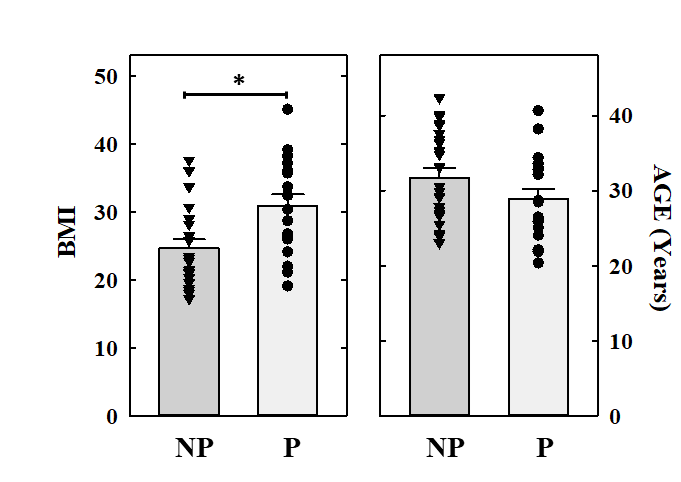
**
